# Supplementary material for: MetaRibo-Seq measures translation in microbiomes
Source: Nat Commun. 2020 Jun 29;11:3268. doi: 10.1038/s41467-020-17081-z (PMC7324362; doi:10.1038/s41467-020-17081-z)
Supplement: Supplementary file 10 — Supplementary Data 7 [file 41467_2020_17081_MOESM10_ESM.zip › File2/Confidence_High_Taxonomy/221529_out.krona.html]

Javascript must be enabled to view this page.

members
magnitude
magnitudeUnassigned
count
unassigned
taxon
rank

221529\_out

86

86
2
superkingdom

phylum
86
1239

class
186801
86

order

SRS016267\_contig\_number\_contig-100\_4700.41971SRS017521\_contig\_number\_contig-100\_8475.54733SRS018427\_contig\_number\_contig-100\_14614.14614SRS058770\_contig\_number\_26215SRS063127\_contig\_number\_720SRS074964\_contig\_number\_contig-100\_21040.21040SRS098571\_contig\_number\_11595SRS1041038\_contig\_number\_9125SRS1055043\_contig\_number\_contig-100\_13231.95376SRS1055099\_contig\_number\_contig-100\_6334.6335SRS147766\_contig\_number\_contig-100\_9094.91010
86
186802
11

1897045

SRS147614\_contig\_number\_812
1
species

12
2109688

SRS012969\_contig\_number\_31580SRS015782\_contig\_number\_39618SRS016132\_contig\_number\_4548SRS045195\_contig\_number\_6342SRS046717\_contig\_number\_8162SRS049995\_contig\_number\_29597SRS077641\_contig\_number\_23610SRS077849\_contig\_number\_11280SRS1041136\_contig\_number\_19682SRS1055034\_contig\_number\_10045SRS144537\_contig\_number\_46031SRS149075\_contig\_number\_contig-100\_24.119253
species

species

SRS019068\_contig\_number\_contig-100\_1540.179748
1898207
1

1

SRS1041039\_contig\_number\_28892
172733
species

family
541000
55

genus
55
946234

55
1193534

SRS012273\_contig\_number\_41490SRS013098\_contig\_number\_43107SRS013965\_contig\_number\_700SRS015190\_contig\_number\_14221SRS015960\_contig\_number\_contig-100\_22692.22692SRS016018\_contig\_number\_contig-100\_28696.28696SRS017916\_contig\_number\_contig-100\_13924.89222SRS019381\_contig\_number\_21478SRS019601\_contig\_number\_27708SRS020233\_contig\_number\_31263SRS020622\_contig\_number\_7184SRS020869\_contig\_number\_23257SRS021219\_contig\_number\_contig-100\_1477.56443SRS022071\_contig\_number\_contig-100\_1241.1241SRS023526\_contig\_number\_35685SRS043411\_contig\_number\_contig-100\_2.108914SRS043768\_contig\_number\_contig-100\_22942.73944SRS047433\_contig\_number\_6136SRS049773\_contig\_number\_35625SRS049959\_contig\_number\_48214SRS050752\_contig\_number\_22976SRS053335\_contig\_number\_17603SRS054956\_contig\_number\_740SRS062701\_contig\_number\_7035SRS063190\_contig\_number\_9306SRS063985\_contig\_number\_contig-100\_11173.50993SRS064557\_contig\_number\_contig-100\_2198.61644SRS065397\_contig\_number\_contig-100\_2569.46000SRS075078\_contig\_number\_16696SRS075878\_contig\_number\_11714SRS076804\_contig\_number\_21266SRS077730\_contig\_number\_contig-100\_3739.46391SRS097889\_contig\_number\_41615SRS097920\_contig\_number\_contig-100\_42097.80460SRS098061\_contig\_number\_contig-100\_19662.19663SRS098655\_contig\_number\_contig-100\_370.63320SRS098717\_contig\_number\_contig-100\_46859.46859SRS098827\_contig\_number\_contig-100\_7715.7715SRS1041137\_contig\_number\_6442SRS1041140\_contig\_number\_18046SRS104197\_contig\_number\_284SRS104311\_contig\_number\_35762SRS105153\_contig\_number\_26534SRS1055022\_contig\_number\_23074SRS140513\_contig\_number\_contig-100\_6116.44681SRS142599\_contig\_number\_30412SRS143342\_contig\_number\_9717SRS143598\_contig\_number\_13399SRS143780\_contig\_number\_30276SRS147022\_contig\_number\_contig-100\_155.93689SRS147425\_contig\_number\_11835SRS147977\_contig\_number\_contig-100\_3568.3569SRS148159\_contig\_number\_28046SRS148721\_contig\_number\_28478SRS149879\_contig\_number\_contig-100\_159.155140
species


SRS019286\_contig\_number\_2685SRS104636\_contig\_number\_28006SRS147139\_contig\_number\_37949SRS148511\_contig\_number\_18478SRS148874\_contig\_number\_16706
1897048
5
species
